# Antibiotic prophylaxis in orthognathic surgery: A complex systematic review

**DOI:** 10.1371/journal.pone.0191161

**Published:** 2018-01-31

**Authors:** Aron Naimi-Akbar, Margareta Hultin, Anna Klinge, Björn Klinge, Sofia Tranæus, Bodil Lund

**Affiliations:** 1 Department of Oral and Maxillofacial Surgery, Karolinska University Hospital, Huddinge, Sweden; 2 Department of Dental Medicine, Division of Cariology, Karolinska Institutet, Stockholm, Sweden; 3 Department of Dental Medicine, Division of Periodontology, Karolinska Institutet, Stockholm, Sweden; 4 Department of Oral and Maxillofacial Surgery and Oral Medicine, Malmo University, Malmo, Sweden; 5 Department of Periodontology, Malmo University, Malmo, Sweden; 6 Health Technology Assessment-Odontology (HTA-O), Malmö University, Malmö, Sweden; 7 Department of Dental Medicine, Division of Oral Maxillofacial Diagnostics and Surgery, Section of Oral Maxillofacial Surgery, Karolinska Institutet, Stockholm, Sweden; Navodaya Dental College and Hospital, INDIA

## Abstract

**Objective:**

In orthognathic surgery, antibiotics are prescribed to reduce the risk of postoperative infection. However, there is lack of consensus over the appropriate drug, the dose and duration of administration. The aim of this complex systematic review was to assess the effect of antibiotics on postoperative infections in orthognathic surgery.

**Methods:**

Both systematic reviews and primary studies were assessed. Medline (OVID), The Cochrane Library (Wiley) and EMBASE (embase.com), PubMed (non-indexed articles) and Health Technology Assessment (HTA) publications were searched. The primary studies were assessed using GRADE and the systematic reviews by AMSTAR.

**Results:**

Screening of abstracts yielded 6 systematic reviews and 36 primary studies warranting full text scrutiny. In total,14 primary studies were assessed for risk of bias. Assessment of the included systematic reviews identified two studies with a moderate risk of bias, due to inclusion in the meta-analyses of primary studies with a high risk of bias. Quality assessment of the primary studies disclosed one with a moderate risk of bias and one with a low risk. The former compared a single dose of antibiotic with 24 hour prophylaxis using the same antibiotic; the latter compared oral and intravenous administration of antibiotics. Given the limited number of acceptable studies, no statistical analysis was undertaken, as it was unlikely to contribute any relevant information.

**Conclusion:**

With respect to antibiotic prophylaxis in orthognathic surgery, most of the studies to date have been poorly conducted and reported. Thus scientific uncertainty remains as to the preferred antibiotic and the optimal duration of administration.

## Introduction

Antibiotic prophylaxis to reduce the risk of postoperative infection after orthognathic surgery is a broadly accepted practice. However, there is lack of consensus with respect to the preferred antibiotic and the dose and duration of administration: the available primary and composite data are contradictory[1–4]. Because of increasing antibiotic resistance, it is important to assess the potential benefits and risks associated with administration of antibiotics. The outcomes of numerous treatment procedures are dependent on effective infection control and treatment, hence antibiotic resistance poses a major threat to modern healthcare,[5]. In some geographic areas antibiotics can no longer be relied on, indicating a post-antibiotic era[6]. The development of antibiotic resistance is closely related to the extent of prescription and rational antibiotic usage is an increasingly important preventive measure [7–9]. The benefit of restrictive antibiotic policies is also evident in low consumption environments, where the levels of resistance are relatively low [10]. It has been shown that short- term use of antibiotics, even a single dose, may select for resistant viridans streptococci. Thus short- term utilisation, such as antibiotic prophylaxis, also needs to be reassessed[11, 12].

A literature review on antibiotic prophylaxis in surgery conducted by The Swedish Council on Health Technology Assessment (SBU) concluded that prolonging antibiotic prophylaxis beyond the day of surgery provides no further benefit [13]. Whether this also applies to orthognathic surgery has not been determined.

Systematic reviews evaluate and summarize the state of knowledge on a defined topic. The methodology is strictly defined, with a reproducible literature search, independent literature analyses and meticulous statistical calculations [14–16]. Failure to adhere to the recommended approach may have untoward consequences: an incorrect conclusion may be drawn, giving the impression of solid evidence. Therefore assessment of primary studies as well as systematic reviews is equally important [17, 18]. A complex systematic review involves quality assessment not only of systematic reviews, but also of the original research. This approach is recommended where reviews published to date show inconsistent results [19].

The aim of the present study was to compare the efficacy of short- and long-term antibiotic prophylaxis in orthognathic surgery, by means of a complex systematic review of the available scientific literature.

## Material and methods

### Objective

The objective of the study was to assess the effect of antibiotics on postoperative infections in orthognathic surgery.

### Criteria for eligible studies

Studies considered eligible for inclusion in this literature review were randomised controlled trials (RCT), systematic reviews and meta-analyses of the outcome of orthognathic surgery undertaken with or without antibiotic cover (no treatment, or placebo). Studies comparing two different antibiotic protocols, or comparing antibiotics with alternative treatments, such as antibacterial rinsing, were also accepted. Exclusion criteria for systematic reviews were a more recent systematic review available by same author, non-systematic review, guidelines, letters, position papers, and consensus statements. Table 1 presents a summary of the predefined study population, intervention, comparison of therapies and the outcome parameters in the eligible studies.

**Table 1:** Parameters of interest in eligible studies.

**P**	Patients subjected to orthognatic surgery
**I**	Antibiotics on day of surgery i.e. short-termed prophylaxis Antibiotics more than day of surgery i.e.”extended” prophylaxis Head-to-head comparison of different antibiotic compounds or regiments
**C**	No antibiotic treatment Placebo Other non-antibiotic treatment e.g. such as antibacterial rinsing Other/comparing antibiotic treatment (alternative compound) Same compound, different dose/duration
**O**	Infection (primary) Quality of life (primary) Pain (primary) Mortality (primary) Osteosynthesis removal Sensory deficiency Patient reported out-come Relapse Length of hospital stay Length of sick leave Health economy Ethical aspects
**Systematic reviews**	**Inclusion criteria** Systematic review Systematic meta-analysis Exclusion criteria for systematic reviews
	**Exclusion criteria** Non-systematic review Guidelines Letter Position paper Consensus statements
**Primary studies**	**Inclusion criteria** English abstract Randomised control trials (RCT)
	**Exclusion criteria** Animal studies In vitro studies Any study type except RCT Lack of follow up

### Search strategies

The initial literature search was undertaken by two of the authors (ANA and AK) and two information specialists at the Karolinska Institutet University Library. The following databases were searched up to October 20, 2015: Medline (OVID), The Cochrane Library (Wiley) and EMBASE (embase.com), PubMed (non-indexed articles). The search terms used for the various databases are summarised in Table 2. The search was initially unfiltered, for the primary studies, then repeated, with a filter, for systematic reviews. Publications by the following Health Technology Assessment (HTA) organisations were searched until October 30, 2015, for projects evaluating the outcome on orthognathic surgery of administration of antibiotics *versus* no treatment or placebo treatment: NICE, http://www.nice.org.uk/; CADTH, http://www.cadth.ca/; CRD database (contains reviews from INAHTA, Cochrane, CRD and NIHR), http://www.crd.york.ac.uk/CRDWeb/; Kunnskapssenteret, http://www.kunnskapssenteret.no/home?language=english; ASERNIP-S http://www.surgeons.org/for-health-professionals/audits-and-surgical-research/asernip-s/publications/.

**Table 2:** Search strategy.

Database	Search strategy	No of references retrieved
Medline (Ovid)	1. exp Orthognathic Surgical Procedures/ 2. exp Orthognathic Surgery/ 3. exp Mandibular Advancement/ 4. exp Osteotomy, Le Fort/ 5. exp Osteotomy, Sagittal Split Ramus/ 6. (orthognat* or sagittal split or genioplast*).tw,kf. 7. (chin adj3 reposition*).tw,kf. 8. (osteotom* adj5 (bimaxillar* or maxillar* or le fort or vertical or ramus or sagittal or bilateral)).tw,kf. 9. (mandibular adj5 osteotom*).tw,kf. 10. or/1-9 11. exp Anti-Bacterial Agents/ 12. exp Antibiotic Prophylaxis/ 13. (antibiot* or anti-bacterial* or antibacterial* or penicillin* or cephalosporin* or cefazolin or cefuroxime or amox?cillin* or clindam?cin or levofloxacin).tw,kf. 14. or/11-13 15. 15. 10 and 14	Systematic reviews: 5 Primary studies: 93
The Cochrane Library (Wiley)	#1: orthognat* or "sagittal split" or genioplast:ti,ab,kw (Word variations have been searched) #2: (chin near/3 reposition*):ti,ab,kw (Word variations have been searched) #3: osteotom* near/5 (bimaxillar* or maxillar* or "le fort" or vertical or ramus or sagittal or bilateral):ti,ab,kw (Word variations have been searched) #4: (mandibular near/5 osteotom*):ti,ab,kw (Word variations have been searched) #: #1 or #2 or #3 or #4 #6: (antibiotic* or cephalosporin* or cefazolin or cefuroxime or amoxicillin* or amoxycillin* or clindamycin or clindamicin* or penicillin* or levofloxacin):ti,ab = 19333 #7: #5 and #6	Systematic reviews: 4 Primary studies: 20
EMBASE (www.embase.com)	#14: #9 AND #13 #13: #10 OR #11 OR #12 #12: antibiotic*:ab,ti OR cephalosporin*:ab,ti OR cefazolin:ab,ti OR cefuroxime:ab,ti OR amoxicillin*:ab,ti OR amoxycillin*:ab,ti ORclindamycin:ab,ti OR clindamycin*:ab,ti OR penicillin*:ab,ti OR levofloxacin:ab,ti #11: 'antibiotic prophylaxis'/de #10: 'antibiotic agent'/exp #9: #1 OR #2 OR #3 OR #4 OR #5 OR #6 OR #7 OR #8 #8: (mandibular NEAR/5 osteotom*):ab,ti #7: (osteotom* NEAR/5 (bimaxillar* OR maxillar* OR 'le fort' OR vertical OR ramus OR sagittal OR bilateral)):ab,ti #6: (chin NEAR/3 reposition*):ab,ti #5: orthognat*:ab,ti OR 'sagittal split':ab,ti OR genioplast*:ab,ti #4: 'mandible osteotomy'/exp #3: 'maxilla osteotomy'/exp #2: 'mandible reconstruction'/exp	Systematic reviews: 40 Primary studies: 298
PubMed* NOT Medline	(((((((antibiotic*[Title/Abstract] OR cephalosporin*[Title/Abstract] OR cefazolin[Title/Abstract] OR cefuroxime[Title/Abstract] OR amoxicillin*[Title/Abstract] OR amoxycillin*[Title/Abstract] OR clindamycin[Title/Abstract] OR clindamycin*[Title/Abstract] OR penicillin*[Title/Abstract] OR levofloxacin[Title/Abstract])))) AND ((((((mandibular[Title/Abstract] AND osteotom*[Title/Abstract]))) OR ((osteotom*[Title/Abstract]) AND (bimaxillar*[Title/Abstract] OR maxillar*[Title/Abstract] OR "le fort"[Title/Abstract] OR vertical[Title/Abstract] OR ramus[Title/Abstract] OR sagittal[Title/Abstract] OR bilateral[Title/Abstract]))) OR ((chin[Title/Abstract] AND reposition*[Title/Abstract]))) OR ((orthognat*[Title/Abstract] OR sagittal split[Title/Abstract] OR genioplast*[Title/Abstract]))))) NOT medline[sb])	Systematic reviews: 1 Primary studies: 9

The reference lists of all the eligible studies were hand-searched for potential complementary trials. Although there was no restriction according to language, retrieved papers in a language other than English were excluded. In order to detect more recent publications, complementary searches were undertaken in PubMed on November 24, 2016 and again on October 31, 2017. These additional searches did not use any filters and all new findings from both primary studies as well as systematic reviews were screened.

### Study selection

Eligible studies were selected according to inclusion/exclusion criteria. ANA (first author) then screened the retrieved list, for initial exclusion of irrelevant publications, based on title. In case of uncertainty, a study was retained until the next selection step: examination of abstracts. The abstracts were read independently, in duplicate, by three reviewers, BL, AK and ST or MH, ANA and BK. Selected primary studies and systematic reviews were read in full-text, in duplicate, by three reviewers each, respectively. Any disagreement during the screening process was resolved by discussion in the project group. Studies excluded at this stage, and the reason for exclusion, were recorded.

### Quality assessment

#### Systematic reviews (SR)

The level of bias for systematic reviews was assessed by AMSTAR [20–22]. The reviews were classified as having low, moderate or high risk of bias according to the criteria shown in Table 3.

**Table 3:** Criteria for low, moderate and high risk of bias according to Mejàre, 2015, based on AMSTAR assessment items.

**Level of bias**	**Criteria**	**Q**
**Low**	Predetermined research question and inclusion criteria established.	1
	A minimum of two independent data extractors and consensus procedure reported.	2
	At least the database MEDLINE/PubMed used. Reproducible search strategy reported.	3
	Included and excluded studies reported. *	4
	Relevant characteristics of included studies stated.	5
	Scientific quality assessed and reported for each included study.	6
	Alignment between scientific quality of included studies and formulating conclusions.	7
	Rational and methods for pooling results reported.	8
	Publication bias estimated. Can be omitted if publication bias was unlikely and not reported.	9
	Conflict of interest stated. This item can be omitted if conflicts of interest were unlikely.	10
**Moderate**	A yes-answer to question 1–7 required.	
**High**	A no-answer to any of the questions listed under moderate risk of bias (except question 1).	

Q, AMSTAR question.

*List of included studies mandatory. The absence of a report of excluded studies is accepted

#### Primary studies

The quality of the included primary studies was assessed according to a protocol for assessment of randomized studies [23].

### Quality of evidence

The scientific quality of the evidence in the primary studies was graded according to GRADE, as high, moderate, low, and very low [24], Table 4.

**Table 4:** Significance of the four levels of evidence.

Quality level	Current definition
**High**	We are very confident that the true effect lies close to that of the estimate of the effect
**Moderate**	We are moderately confident in the effect estimate: The true effect is likely to be close to the estimate of the effect, but there is a possibility that it is substantially different
**Low**	Our confidence in the effect estimate is limited: The true effect may be substantially different from the estimate of the effect
**Very low**	We have very little confidence in the effect estimate: The true effect is likely to be substantially different from the estimate of effect

### Data extraction

#### Systematic reviews

Data extracted from the systematic reviews covered objectives, main results, authors’ estimated level of evidence, and knowledgegaps according to authors.

#### Primary studies

The following data were extracted from the primary studies: number of patients included, age, gender distribution, length of follow-up, type of intervention and type of control treatment and relevant treatment outcomes.

### Assessment of publication bias and heterogeneity (primary studies)

Not applicable: there were insufficient studies for meta-analysis.

## Results

### Literature search and study selection

**Fig 1:**
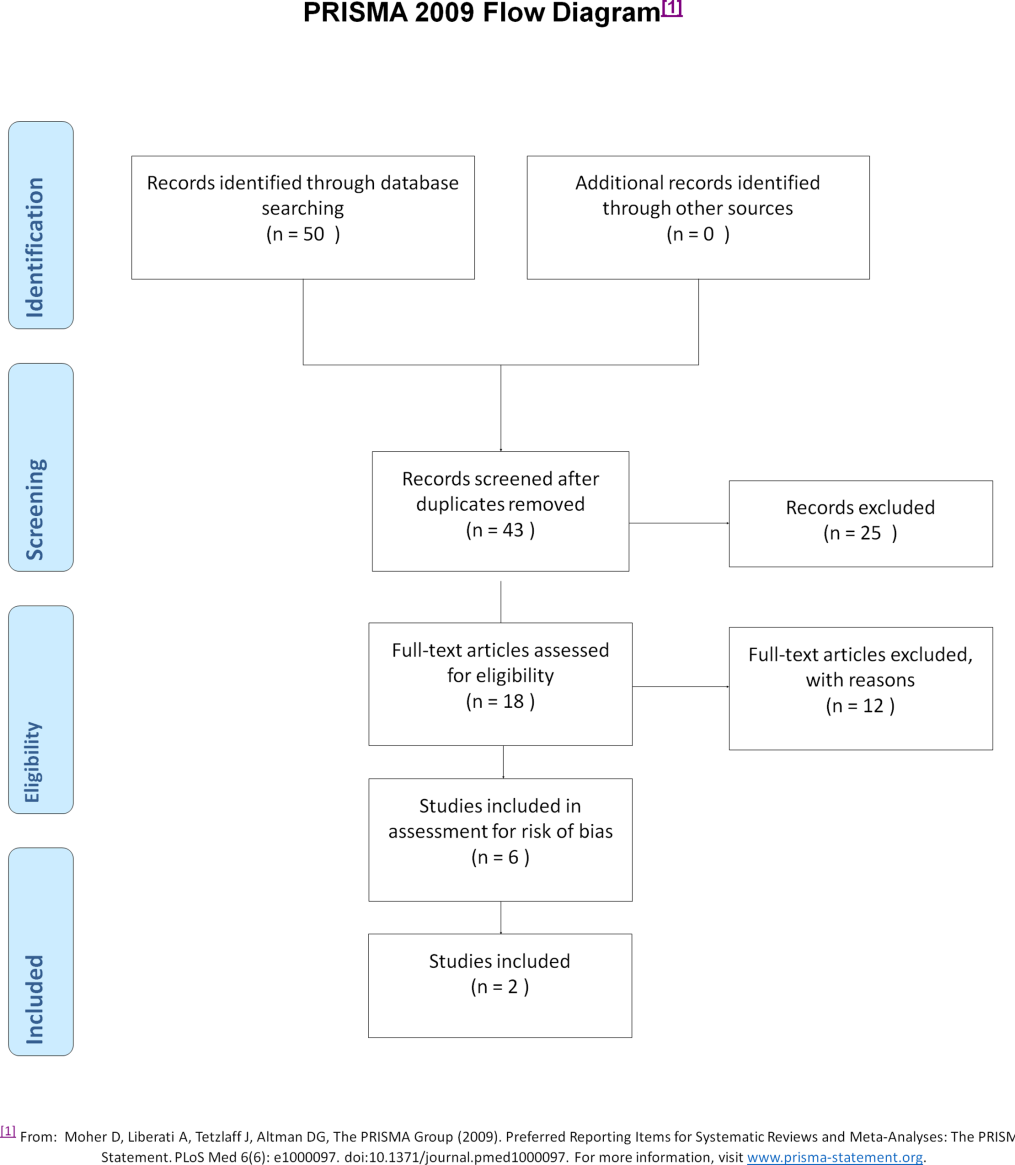
Flow chart, systematic reviews.

**Fig 2:**
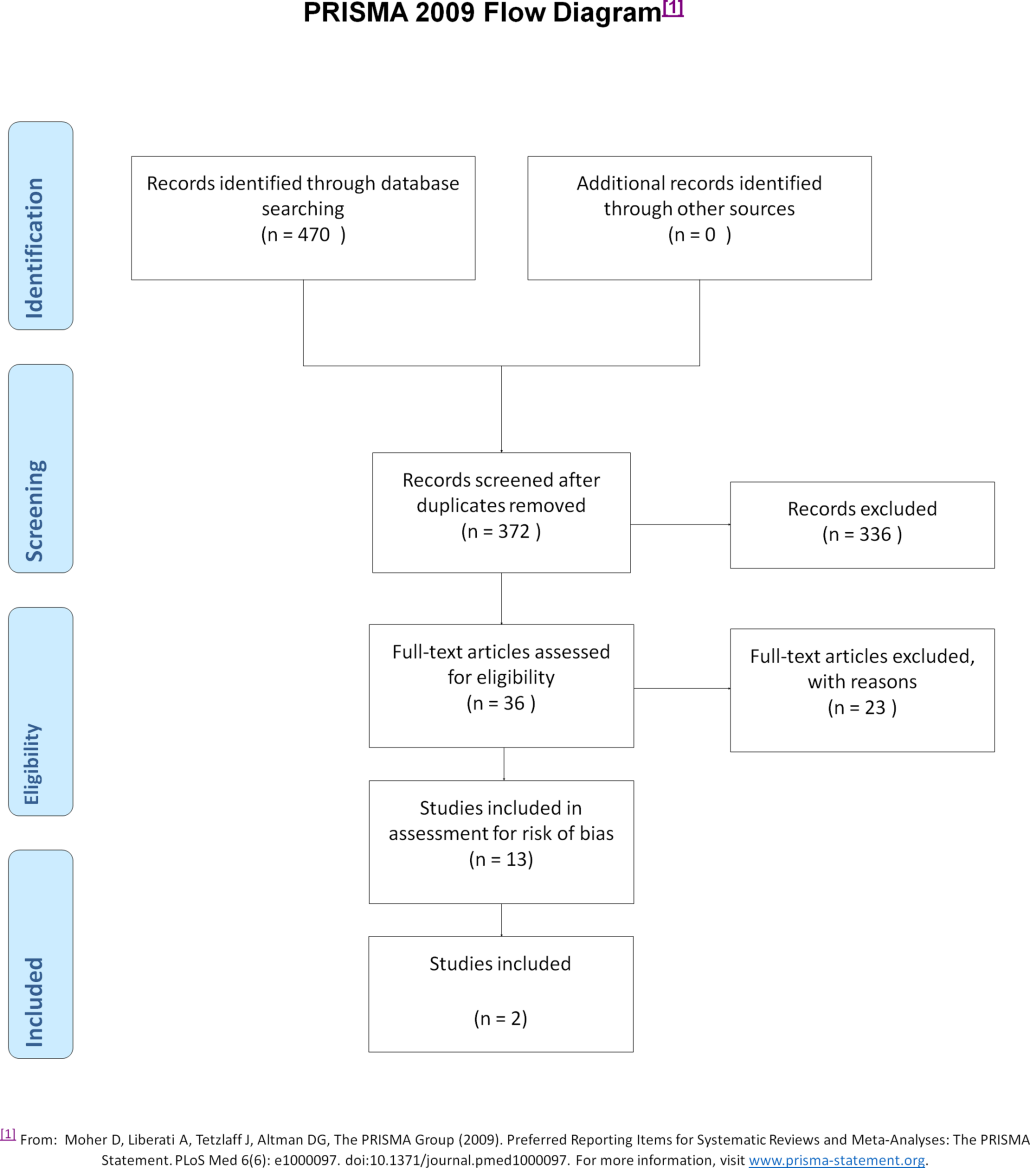
Flow chart, primary studies.

The search for systematic reviews yielded 50 articles: 43 remained after exclusion of duplicates. The search strategy for primary studies yielded 470 articles: after exclusion of duplicates, 372 remained. The number of studies retrieved from each data base search is shown in Table 2. The search of publications by HTA organisations failed to identify any further studies. Flow-charts of the screening process for systematic reviews and primary studies are described in Figs 1 and 2, respectively.

The additional search on November 24, 2016 added 56 new articles and the search on October 31, 2017 added 38 new articles: all were screened for both systematic reviews and primary studies.

#### Systematic reviews

Screening of abstracts yielded six SR warranting retrieval of the fulltext for scrutiny. The additional searches did not yield any further systematic reviews for inclusion.

#### Primary studies

In all, 36 primary studies were read in full-text, 23 of which were excluded, leaving 13primary studies eligible for quality assessment. Table 5 presents primary studies read in full text but then excluded from quality assessment, along with the reason for exclusion. The additional search on November 24, 2016 yielded one more study to undergo quality assessment. Thus in all, 14 studies were assessed.

**Table 5:** Excluded primary studies read in full text.

Reference	Reason for exclusion
Barrier A, Breton P, Girard R, Dubost J, Bouletreau P. Surgical site infections in orthognathic surgery and risk factors associated. Rev Stomatol Chir Maxillofac. 2009;110(3):127–34. Les infections du site operatoire en chirurgie orthognathique et leurs facteurs de risque.	Not in English
Bystedt H, Josefsson K, Nord CE. Ecological effects of penicillin prophylaxis in orthognathic surgery. Int J Oral Maxillofac Surg. 1987;16(5):559–65.	Not RCT
Deffez JP, Wierzbicky N, Allain P. Comparative efficacy of preoperative potentialization of immunity defenses and postoperative antibiotic therapy. Rev Stomatol Chir Maxillofac. 1985;86(5):320–6.	Not in English
Deffez JP, Wierzbicky N, Allain P, Brethaux J, Lelay C, Themar P. [Preoperative potentiation of immune defenses or postoperative antibiotic therapy?]. Rev Stomatol Chir Maxillofac. 1985;86(5):320–6. Potentialisation pre-operatoire des defenses immunitaires ou antibiotherapie post-operatoire?	Not in English
Dodson TB, Halperin LR. Prophylactic antibiotics reduce infectious complications of orthognathic surgery. Evidence-based Dentistry. 2000; 2(3):[66 p.].	Commentary
Dunham J. Cosmetic maxillofacial surgery. Journal of Oral and Maxillofacial Surgery. 2012;70(11):e310-e30.	Not RCT
Dunham J. Reconstructive surgery. Journal of Oral and Maxillofacial Surgery. 2012;70(11):e272-e309.	Not RCT
Fridrich KL. Preoperative antibiotic prophylaxis in orthognathic surgery: A randomized, double-blind, and placebo-controlled clinical study—Discussion. Journal of Oral and Maxillofacial Surgery. 1999;57(12):1406–7.	Commentary
Harrell L, Shetty V. Extended antibiotic therapy may reduce risk of infection following orthognathic surgery. The journal of evidence-based dental practice. 2012;12(3):144–5. Epub 2012/09/01.	Not RCT
Igawa HH, Sugihara T, Yoshida T, Kawashima K, Ohura T. Penetration of flomoxef into human maxillary and mandibular bones. Scandinavian Journal of Plastic and Reconstructive Surgery and Hand Surgery. 1995;29(3):259–62	Not RCT
Jourde J. Use of oxytetracycline and doxycycline in maxillofacial surgery. Lyon Medical. 1973;230(14):245–7.	Not in English
Liebermann B, Röthig W, Kaufhold W. Perioperative antimicrobial prophylaxis in Obwegeser-Dal Pont surgery for prognathism. Deutsche Zeitschrift für Mund-, Kiefer- und Gesichts-Chirurgie. 1990;14(6):418–23.	Not RCT
Martis C, Karabouta I. Infection after orthognathic surgery, with and without preventive antibiotics. Int J Oral Surg. 1984;13(6):490–4.	Not RCT
Peterson LJ, Booth DF. Efficacy of antibiotic prophylaxis in intraoral orthognathic surgery. J Oral Surg. 1976;34(12):1088–91.	Not RCT
Salmeron-Escobar JI, del Amo-Fernandez de Velasco A. Antibiotic prophylaxis in Oral and Maxillofacial Surgery. Med Oral Patol Oral Cir Bucal. 2006;11(3):E292-6.	Not RCT
Samman N, Cheung LK. Antibiotic prophylaxis for orthognathic surgery: a prospective trial of four penicillin regimes [abstract]. J Craniomaxillofac Surg. 1996; 24(Suppl 1).	Congress abstract
Schubert J, Schafer R. [Results of perioperative antibiotic prophylaxis in orthognathic surgery]. Dtsch Z Mund Kiefer Gesichtschir. 1990;14(2):96–8. Erfahrungen mit perioperativer Antibiotikaprophylaxe bei Dysgnathieoperationen.	Not in English
Schubert J, Schäfer R. Results of perioperative antibiotic prophylaxis in orthognathic surgery. Deutsche Zeitschrift für Mund-, Kiefer- und Gesichts-Chirurgie. 1990;14(2):96–8.	Not in English
Simons JP, Johnson JT, Yu VL, Vickers RM, Gooding WE, Myers EN, et al. The role of topical antibiotic prophylaxis in patients undergoing contaminated head and neck surgery with flap reconstruction. Laryngoscope. 2001;111(2):329–35.	Not correct research question
Spaey YJ, Bettens RM, Mommaerts MY, Adriaens J, Van Landuyt HW, Abeloos JV, et al. A prospective study on infectious complications in orthognathic surgery. J Craniomaxillofac Surg. 2005;33(1):24–9.	Not RCT
Yamashita N, Matsuno T, Miyai T, Arai C, Adachi M, Satoh T. Study on diachronic changes of inflammatory cytokines and perioperative management in orthognathic surgery. Oral Therapeutics and Pharmacology. 2007;26(2):37–43.	Not in English
Yrastorza JA. Indications for antibiotics in orthognathic surgery. J Oral Surg. 1976;34(6):514–6.	Not RCT
Zijderveld SA, Smeele LE. Preoperative antibiotic prophylaxis in orthognathic surgery: a randomised double-blind and placebo-controlled clinical trial [abstract]. Br-Journal of Oral and Maxillofacial Surgery. 1998; 36.	Congress abstract

### Quality assessment and data extraction of systematic reviews and meta-analyses

The quality assessment of the included systematic reviews identified two studies assessed as having a moderate risk of bias because of the inclusion in the meta-analyses of primary studies at high or unclear risk of bias [1, 25], see Table 6. Four systematic reviews were considered to be at high risk of bias [2, 3, 26, 27] (Table 7). The main shortcomings were inadequate consideration of quality assessment when formulating conclusions (n = 4), inadequate or unclear search strategy (n = 3), failure to include the characteristics and results of included studies (n = 3), study selection and data extraction had not been undertaken by two independent reviewers (n = 2), and failure to account for included and excluded studies (n = 2). No studies were considered to be at low risk of bias.

**Table 6:** Systematic reviews at moderate risk of bias.

Author Year	Objectives	Main results*	Estimated level of evidence*	Knowledge/ Knowledge gaps*	Level of risk of bias Comments
**Brignardello-Petersenet al. 2015**	Assess the effect of antibiotic prophylaxis in orthognathic surgery for prevention of SSI.	1) Long-term antibiotic prophylaxis probably reduces the risk of SSI. 2) Effects of short-term versus single dose prophylaxis are uncertain.n = 11	1) Moderate quality of evidence for long-term prophylaxis 2) Low quality of evidence for short-term prophylaxis	Antibiotics seem beneficial for reducing SSI.Preferred type of compound unknown.	Moderate.Studies with high risk of bias included in the meta-analyses.
**Tan et al.2011**	Investigate the efficacy of prophylactic antibiotics for prevention of SSI after orthognathic surgery.	1) Antibiotics reduce rate of SSI. 2) No significant difference between long- and short-term prophylaxis.n = 5	Low risk of bias concerning blinding (n = 4) and sequence generation (n = 4).Unclear risk for other sources of bias (n = 5).	Antibiotic prophylaxis significantly reduces SSI.No further benefit of extended antibiotic prophylaxis.	ModerateStudies with high risk of bias included in meta-analyses. Meta-analysis fixed model used despite heterogeneous studies

*According to authors.

Abbreviations: SSI, surgical site infections; n, number of studies included in meta-analyses

**Table 7:** Systematic reviews excluded due to high risk of bias.

Reference	No answer to AMSTAR question number
Ariyan et al. Antibiotic prophylaxis for preventing surgical site infection in plastic surgery: An evidence based consensus conference statement from American association of plastic surgeons. Plast Reconstr Surg 2015;135:1723–39.	1, 3–4, 6–8, 10
Danda et al. Effectiveness of postoperative antibiotics in orthognathic surgery: A meta-analysis. J oral maxillofac surg 2011;69:2650–56.	1–2, 7–9, 11
Kreutzer et al. Current evidence regarding prophylactic antibiotics in head and neck and maxillofacial surgery. Biomed research international 2014:1–7.	1–3, 5–10
Oomens et al. Prescribing antibiotic prophylaxis in orthognathic surgery: a systematic review. Int j oral maxillofac surg 2014;43:725–31.	1, 6, 8, 10

### Quality assessment and data extraction of primary studies

**Fig 3:**
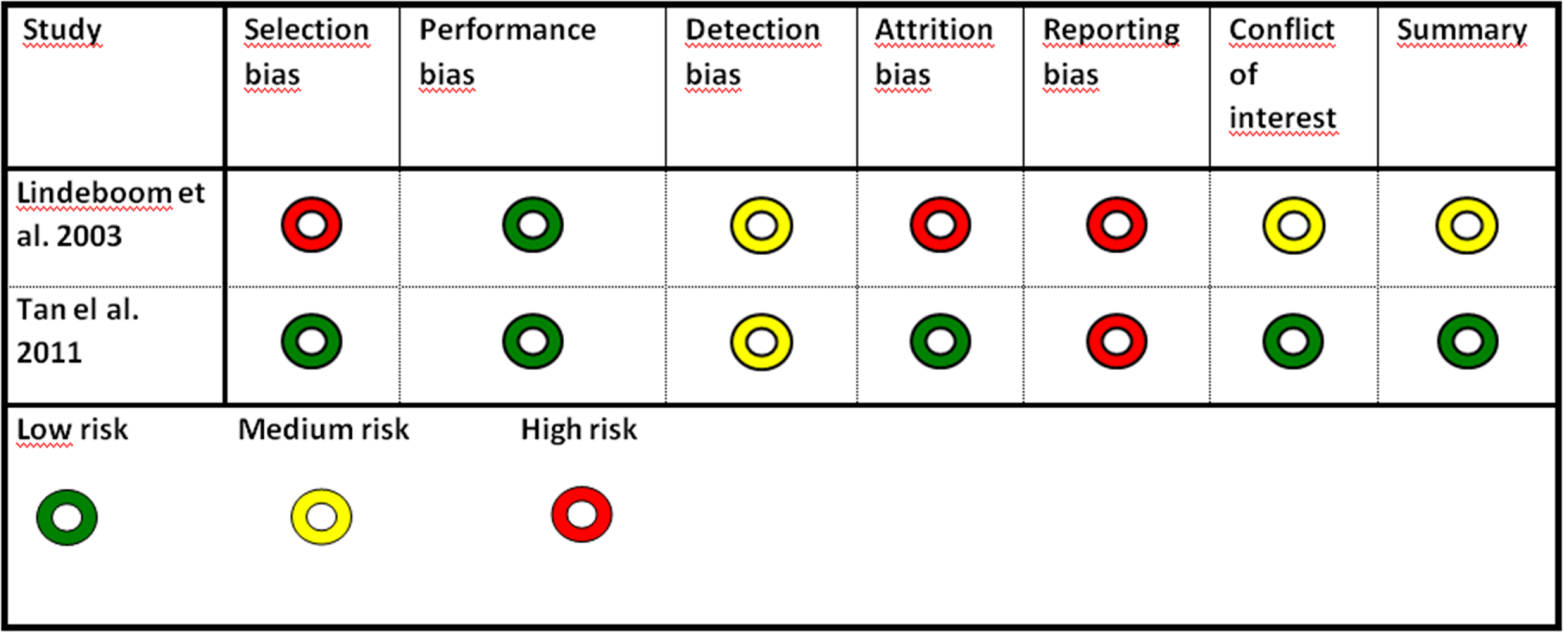
Methodological assessment of included studies.

The quality assessment identified one study with a moderate risk of bias [28] (Lindeboom et al. 2003) and one with a low risk of bias [29] (Tan et al. 2011). A high risk of bias was found in 12 studies. The study with a moderate risk of bias compared a single dose of antibiotic with 24 hour prophylaxis using the same compound. The study with a low risk of bias compared oral and intravenous administration of antibiotics (Table 8). The type of intervention and study population characteristics in studies classified as being at low or moderate risk of bias are shown in Table 8. The most common reason for high risk of bias was a poorly described, unclear randomization process (Table 9). Table 9 shows the eligible studies excluded due to a high risk of bias. The outcomes of the included primary studies with low or moderate risk of bias are presented in Table 10. Fig 3 presents a summary of the quality assessment of the included studies with low or moderate risk of bias.

**Table 8:** Characteristics and quality assessment of included primary studies with low or moderate risk of bias.

Author Year Country	Population	Study period	Intervention	Control	Risk of bias Comments
**Lindeboom et al.** **2003** **Netherlands**	n: 70 Age: 19–54 years Gender (m/f):52/18 Surgery: Bilateral sagittal ramus osteotomy of the mandible Smokers: not reported	3 months	600 mg intravenous clindamycin 15 minutes prior to surgery–intravenous placebo solution 6 hourly for 24 hours postoperatively	600 mg intravenous clindamycin 15 minutes prior to surgery –600 mg intravenous clindamycin 6 hourly for 24 hours postoperatively	Moderate risk of bias No published study protocol No published distribution of base line data between study groups No reports of loss to follow-up
**Tan et al. 2011** **China**	n: 42 Age: 18–40 years Gender (m/f): 28/14 Surgery: Bimaxillary orthognathic surgery Smokers: none (exclusion criteria)	6 weeks	1 g intravenous ampicillin prior to surgery, 500 mg ampicillin every 6 hours during operation 500 mg oral amoxicillin three times a day and intravenousplacebo four times a day, both for two days postoperatively 500 mg oral amoxicillin three times daily for the next three days	1 g intravenous ampicillin prior to surgery, 500 mg ampicillin every 6 hours during operation 1 g intravenous ampicillin four times daily andoral placebo three times a day, both for two days postoperatively 500 mg oral amoxicillin three times daily for the next three days	Low risk of bias No published study protocol No reports of loss to follow-up

Abbreviations: n, number of patients; m/f, male/female; g, gram; h, hour; preop, preoperative; postop, postoperative; mg, milligram.

**Table 9:** Included studies with high risk of bias.

Author	Main reason for high risk of bias
Baqain ZH, Hyde N, Patrikidou A, Harris M. Antibiotic prophylaxis for orthognathic surgery: a prospective, randomised clinical trial. Br J Oral Maxillofac Surg. 2004;42(6):506–10.	Unclear outcome measure
Bentley KC, Head TW, Aiello GA. Antibiotic prophylaxis in orthognathic surgery: a 1-day versus 5-day regimen. J Oral Maxillofac Surg. 1999;57(3):226–30; discussion 30–2.	Unclear randomization process
Danda AK, Wahab A, Narayanan V, Siddareddi A. Single-dose versus single-day antibiotic prophylaxis for orthognathic surgery: a prospective, randomized, double-blind clinical study. J Oral Maxillofac Surg. 2010;68(2):344–6.	Unclear randomization process, unclear report of methods
Davis CM, Gregoire CE, Davis I, Steeves TW. Prevalence of Surgical Site Infections Following Orthognathic Surgery: A Double-Blind, Randomized Controlled Trial on a 3-Day Versus 1-Day Postoperative Antibiotic Regimen. J Oral Maxillofac Surg. 2016:3.	High drop-out
Eshghpour M, Khajavi A, Bagheri M, Banihashemi E. Value of prophylactic postoperative antibiotic therapy after bimaxillary orthognathic surgery: a clinical trial. Iranian journal of otorhinolaryngology. 2014;26(77):207–10. Epub 2014/10/17.	Unclear randomization process and blinding
Fridrich KL, Partnoy BE, Zeitler DL. Prospective analysis of antibiotic prophylaxis for orthognathic surgery. Int J Adult Orthodon Orthognath Surg. 1994;9(2):129–31.	Unclear randomization process and blinding
Jansisyanont P, Sessirisombat S, Sastravaha P, Bamroong P. Antibiotic prophylaxis for orthognathic surgery: a prospective, comparative, randomized study between amoxicillin-clavulanic acid and penicillin. J Med Assoc Thai. 2008;91(11):1726–31.	Unclear randomization process and blinding
Kang SH, Yoo JH, Yi CK. The efficacy of postoperative prophylactic antibiotics in orthognathic surgery: a prospective study in Le Fort I osteotomy and bilateral intraoral vertical ramus osteotomy. Yonsei Med J. 2009;50(1):55–9.	Unclear blinding and follow-up procedure
Ruggles JE, Hann JR. Antibiotic prophylaxis in intraoral orthognathic surgery. J Oral Maxillofac Surg. 1984;42(12):797–801.	No baseline data reported, unclear follow-up procedure
Wahab PU, Narayanan V, Nathan S, Madhulaxmi. Antibiotic prophylaxis for bilateral sagittal split osteotomies: a randomized, double-blind clinical study. Int J Oral Maxillofac Surg. 2013;42(3):352–5.	Unclear randomization process and blinding
Yoda T, Sakai E, Harada K, Mori M, Sakamoto I, Enomoto S. A randomized prospective study of oral versus intravenous antibiotic prophylaxis against postoperative infection after sagittal split ramus osteotomy of the mandible. Chemotherapy. 2000;46(6):438–44.	Unclear randomization process
Zijderveld SA, Smeele LE, Kostense PJ, Bram Tuinztng D. Preoperative antibiotic prophylaxis in orthognathic surgery: A randomized, double-blind, and placebo-controlled clinical study. Journal of Oral and Maxillofacial Surgery. 1999;57(12):1403–6.	Unclear randomization process and outcome measure

**Table 10:** Outcome of included primary studies with low or moderate risk of bias.

Author Year Country	Outcome				
	Intervention		Control		Result
	Post operative infection	No.	Post operative infection	No.	
**Lindeboom et al.** **2003** **Netherlands**	clindamycin 600 mg 1 hour preop 2 patients with postoperative infection	35	clindamycin 24 hour 1 patients with postoperative infection	35	RR– 2.00
**Tan et al.** **2011** **China**	Oral amoxicillin 500mg three times daily for the first two days 3 patients with postoperative infection	21	1g ampicillin four times daily for the first two days 6 patients with postoperative infection	21	RR– 0.50

Abbreviations: No, number of patients; g, gram; preop, preoperative; hr, hour; postop, postoperative, mg, milligram, RR, risk ratio.

Because of the limited material, no statistical analysis was undertaken.

## Discussion

This complex systematic review of prophylactic antibiotics in orthognathic surgery identified no systematic reviews at low risk of bias and two at moderate risk. The latter had somewhat discrepant results. Although both studies concluded that the use of antibiotics in orthognathic surgery was beneficial, Tan et al. concluded that prolonging antibiotic cover offered no further benefit and Brignardello-Petersen suggested the opposite.

The main shortcoming in both of these systematic reviews is the inclusion in their meta-analyses of studies at either high risk [24], or unclear risk of bias [1]. There is an implied risk that the scientific shortcomings of the included primary studies may unduly influence the major conclusions of the systematic review. As the reader of the systematic review generally accepts the author’s assessments of the included primary studies, such misuse of statistics is particularly problematic. The experienced reader can fairly easily detect the inclusion in the meta-analysis of primary studies with high risk of bias. Applying the description “unclear risk of bias” when lack of information about material and methods precludes assessment of scientific quality is *per se* acceptable. However, such studies should not be included in the meta-analysis as equivalent to studies at either low or moderate risk of bias: this is speculative and misleading. Such a systematic review is clearly at moderate to high risk of bias. In summary, this highlights the importance of meticulous and stringent quality assessment of reviews before drawing conclusions, especially when the conclusions are relevant to clinical practice.

The review of primary studies identified two studies, with different aims, at low or moderate risk of bias. These two studies have also been included in previous systematic reviews presented in this complex systematic review. There were no significant differences between any of the groups in the included studies, and the data were so limited that no conclusions should be drawn about potential beneficial effects in any treatment group. The differing aims of the two studies precluded meta-analysis.

A disappointing finding in this complex systematic review is that twelve studies could have been included in the results if they had met the required standards for randomized controlled trials. Despite the availability of guidelines for reporting RCTs, suboptimal study design, methods, and interpretation of results emerged as major weaknesses[30]. The findings presented following these shortcomings might not be guidelines for clinicians making decisions about the proper use of antibiotics in conjunction with orthognathic surgery, but more of a signal to researchers about the proper conduct of clinical trials and adherence to guidelines when reporting their results.

Failure to adhere to a proper study protocol may also raise ethical issues, implying not only that the inexperienced reader might be misled by the results and that economic resources were being used inappropriately, but also that the subjects had undergone participation in a trial which lacked the potential to improve scientific knowledge.

### Conclusion

In the field of orthognathic surgery, most studies of antibiotic prophylaxis to date have been poorly performed and reported. Scientific uncertainty remains with respect to the preferred antibiotic compound and the optimal range of the prophylaxis.

## Supporting information

S1 PRISMA Checklist PRISMA checklist. 2009 checklist for reporting of systematic reviews.
(DOC)
